# Supplementary figures and images for: RNA polymerase II-mediated rDNA transcription mediates rDNA copy number expansion in Drosophila
Source: PLoS Genet. 2024 May 17;20(5):e1011136. doi: 10.1371/journal.pgen.1011136 (PMC11139327; doi:10.1371/journal.pgen.1011136)

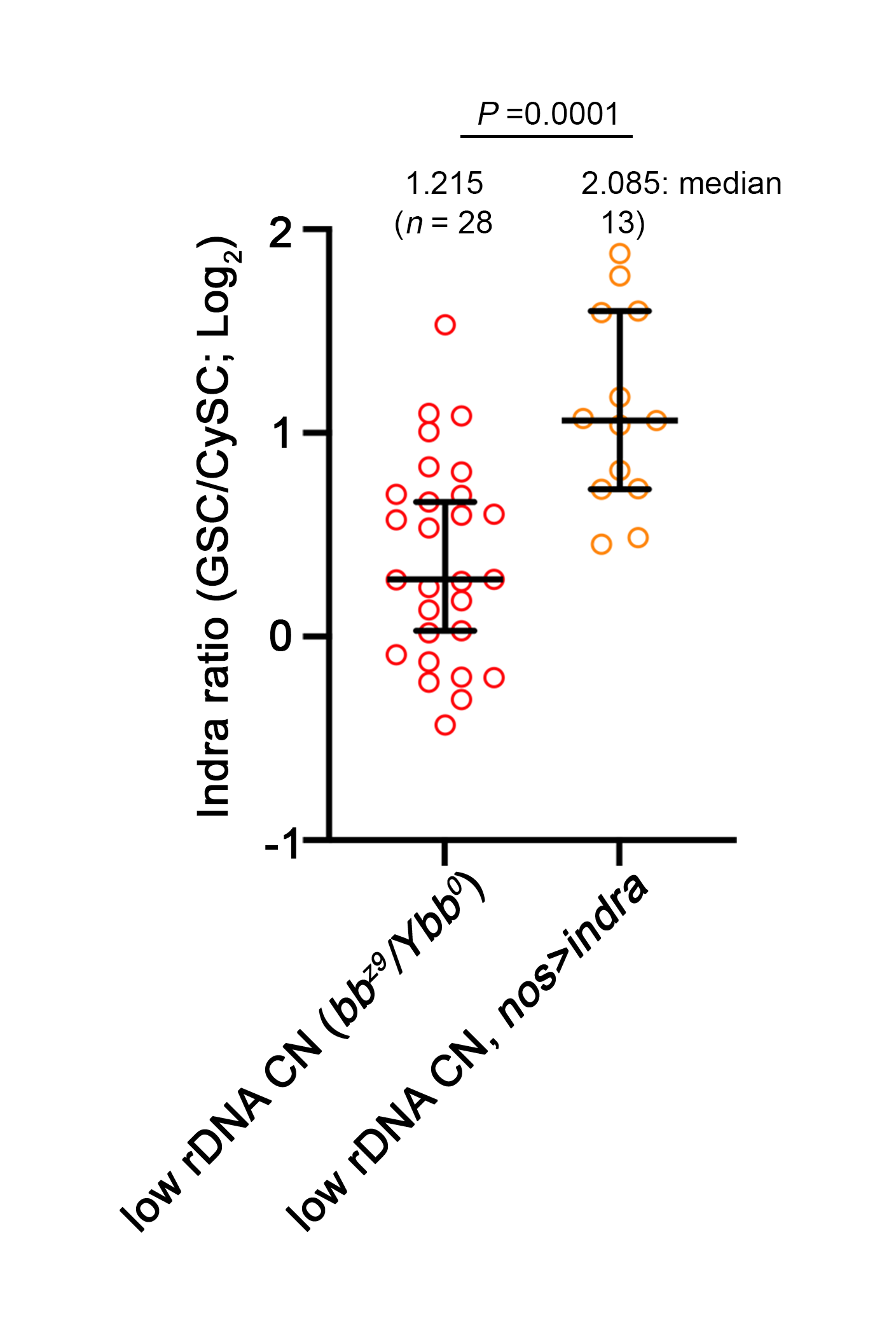

Supplement: S1 Fig — Indra amount in GSCs relative to CySCs under low rDNA condition without or with Indra overexpression in GSCs. Because CySCs do not express Indra transgene, it serves as an appropriate denominator to determine the level of Indra overexpression in GSCs. n = number of GSCs scored. P value, two-tailed Mann-Whitney test. The error bar indicates the median with a 95% CI. (TIF) [file pgen.1011136.s001.tif]

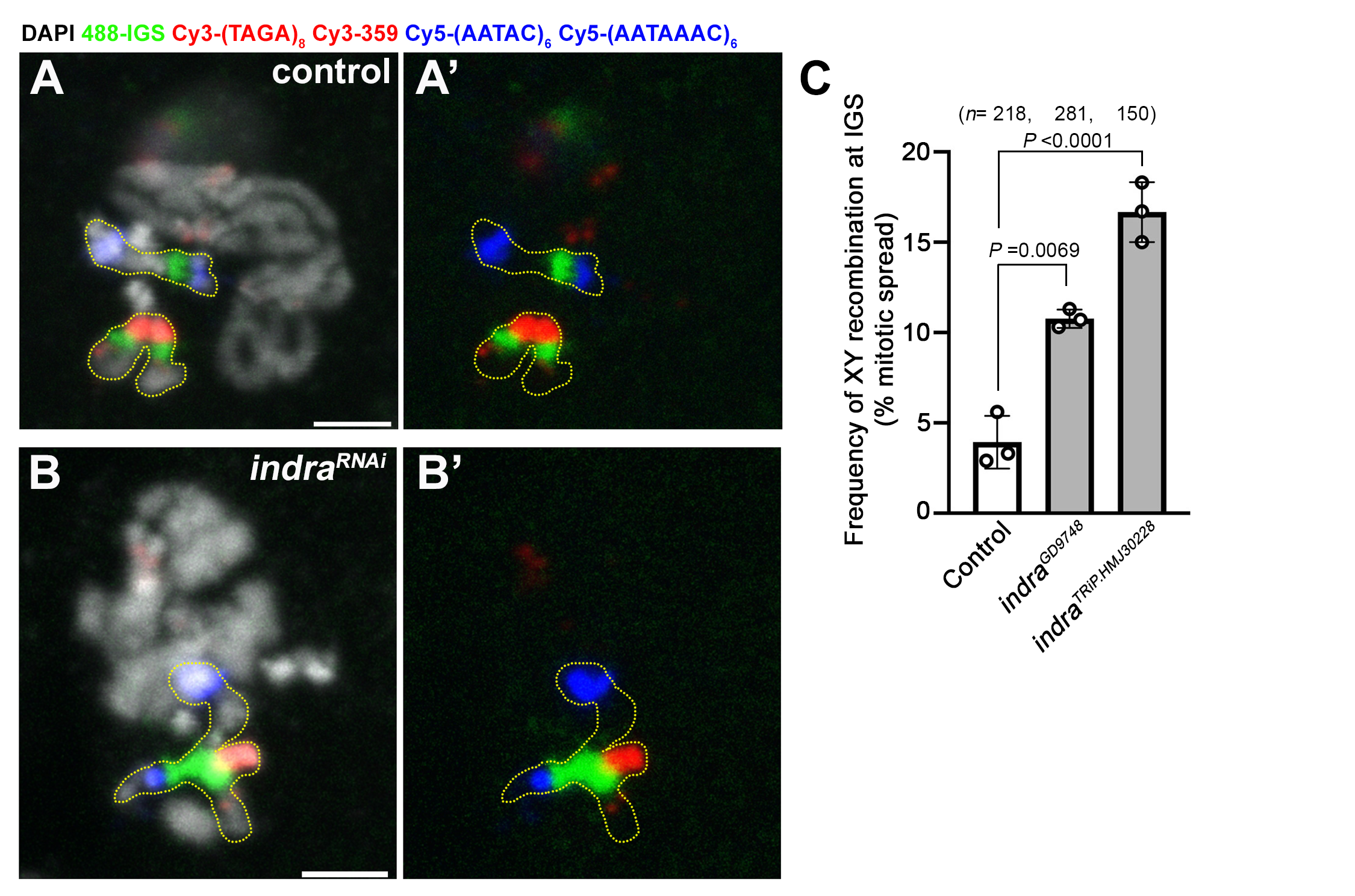

Supplement: S2 Fig — A, B) DNA FISH on the mitotic chromosome spread from control (A) or indraRNAi (B) early germ cells. FISH probes: Alexa488-IGS (rDNA loci on X and Y chromosomes, green); Cy3-(TAGA)8, Cy3-359 (X chromosome, red); Cy5-(AATAC)6, Cy5-(AATAAAC)6 (Y chromosome, blue). Bar: 2.5μm. C) Frequency of early germ cells that exhibit recombination at rDNA upon indra knockdown. (nos-gal4>UAS-indraGD9748, UAS-Dcr-2, and tub-gal80ts, nos-gal4ΔVP16>UAS-indraTRiP.HMJ30228). n = number of mitotic spreads of early germ cells scored. P values, two-sided Fisher’s exact test. The error bar indicates the mean with SD. (TIF) [file pgen.1011136.s002.tif]

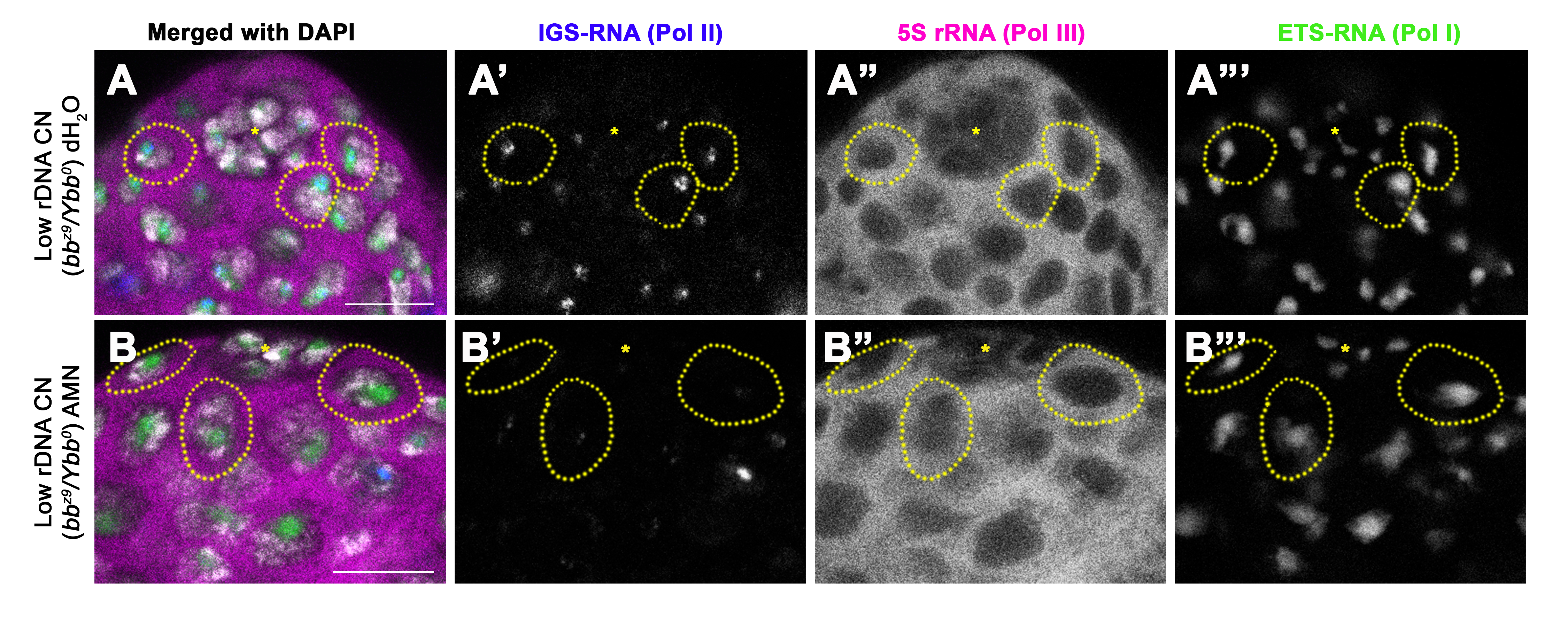

Supplement: S3 Fig — A, B) in situ hybridization of IGS (A’, B’), 5S rRNA (A”, B”), and ETS (A’”, B”’) transcripts under low rDNA conditions treated with dH2O (A-A”‘) or α-amanitin (AMN, B-B”’). IGS was specifically sensitive to Pol II inhibition by α-amanitin, whereas 5S rDNA (transcribed by Pol III) and ETS (transcribed by Pol I) were not affected. Bar: 10 μm. (TIF) [file pgen.1011136.s003.tif]

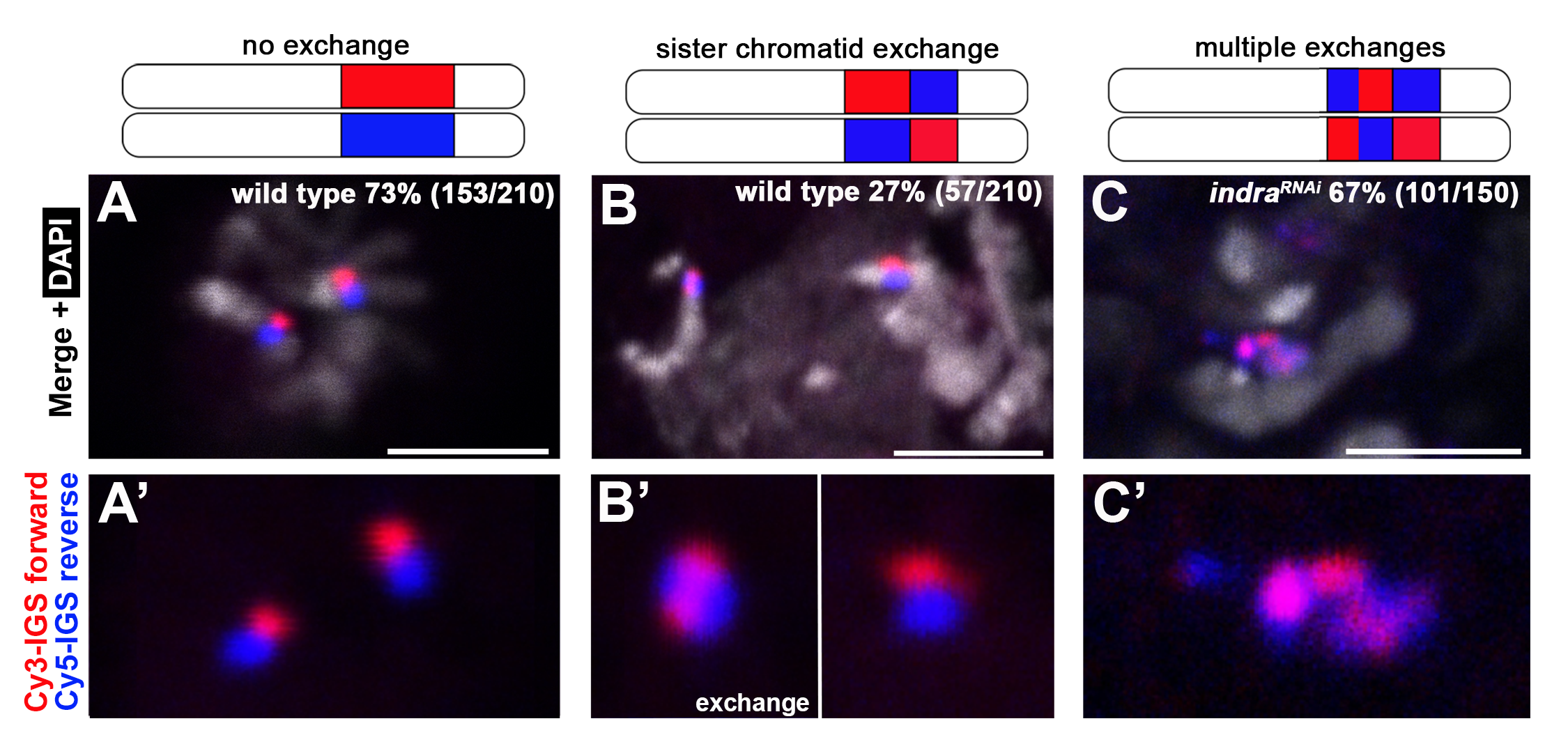

Supplement: S4 Fig — A-C) CO-FISH on mitotic spread of germline stem cells (GSCs) in control (A, B) and indraRNAi (C). In control, ‘blue strand’ and ‘red strand’, representing each sister chromatid, were juxtaposed and no sister chromatid exchange was observed in 73% of GSCs (A). In the remaining 27% of cases (B), one of rDNA loci (X or Y) exhibited one sister chromatid exchange. In indraRNAi, we often (67%) observed multiple sister chromatid exchanges. This may also involve homologous recombination between X and Y rDNA loci, as indicated by the data shown in S1 Fig. Bar: 5μm. Note that upd-overexpression condition was used to enrich GSCs. (TIF) [file pgen.1011136.s004.tif]
