# Supplementary material for: RNA polymerase II-mediated rDNA transcription mediates rDNA copy number expansion in Drosophila
Source: PLoS Genet. 2024 May 17;20(5):e1011136. doi: 10.1371/journal.pgen.1011136 (PMC11139327; doi:10.1371/journal.pgen.1011136)
Supplement: S1 Table — (DOCX) [file pgen.1011136.s005.docx]

**S1 Table. Probe sequences for RNA FISH, DNA FISH, and CO-FISH**

| **Probe target** | **5’-sequence-3’** | **Source or reference** | **Related figure** |
| --- | --- | --- | --- |
| 240-bp IGS | **Alexa488**-TCCATTCACTAAAATGGCTTTTCTCTATAATACTTAGAGAATATGGGAATATTTCAACATTTTTCACT | (1) | Fig 1C-1E,  Fig 2A-2D,  Fig 7A-7C  S1A-S1C Fig |
| 240-bp IGS  (forward) | **Each oligo was labeled with Cy3 on 5’ side**  TTGCCGACCTCTCATATTGTTCAAAACGTATGTGTTCATA, ATTTTGGCAATTATATGAGTAAATTAAATCATATACATAT, GAAAATTAATATTTATTATGTGTATAAGTGAAAAATGTTG,  CATTTTAGTGAATGGATATAGTAGTGTAAGCTAGCTGTTC, AGTGAAAAATGTTGAAATATTCCCATATTCTCTAAGTATTATAGAGAAAAGCCATTTTAGTGAATGGA, TACGACAGAGGGTTCAAAAACTACTATAGGTAGGCAGTGG | This study | S2A-S2C Fig |
| 240-bp IGS  (reverse) | **Each oligo was labeled with Cy5 on 5’ side**  CCACTGCCTACCTATAGTAGTTTTTGAACCCTCTGTCGTA, GAACAGCTAGCTTACACTACTATATCCATTCACTAAAATG, CTTTTCTCTATAATACTTAGAGAATATGGGAATATTTCAA, CAACATTTTTCACTTATACACATAATAAATATTAATTTTC, ATATGTATATGATTTAATTTACTCATATAATTGCCAAAAT, TATGAACACATACGTTTTGAACAATATGAGAGGTCGGCAA | This study | S2A-S2C Fig |
| ITS | **Cy5**-ATTAGCCAACGTATGCCCATAACTA | (2) | Fig 1C-1E |
| ETS | **Cy3**-GATAATAATATACTTTATTATAAGTAACCAATATA | This study | Fig 1E,  Fig 2A-2D,  Fig 7A-7C |
| (TAGA)_n_ | **Cy3**-(TAGA)_8_ | (3) | S1A-S1C Fig |
| 359-bp | **Cy3**-CCACATTTTGCAAATTTTGATGACCCCCCTCCTTACAAAAAATGCG | (4) | S1A-S1C Fig |
| (AATAC)_n_ | **Cy5**-(GTATT)_6_ | (4) | S1A-S1C Fig |
| (AATAAAC)_n_ | **Cy5**-(GTTTATT)_6_ | (3) | S1A-S1C Fig |

**References:**

1. Lu KL, Nelson JO, Watase GJ, Warsinger-Pepe N, Yamashita YM. Transgenerational dynamics of rDNA copy number in Drosophila male germline stem cells. Elife. 2018;7.

2. Nelson JO, Slicko A, Yamashita YM. The retrotransposon R2 maintains Drosophila ribosomal DNA repeats. Proc Natl Acad Sci U S A. 2023;120(23):e2221613120.

3. Jagannathan M, Warsinger-Pepe N, Watase GJ, Yamashita YM. Comparative Analysis of Satellite DNA in the Drosophila melanogaster Species Complex. G3 (Bethesda). 2017;7(2):693-704.

4. Yadlapalli S, Yamashita YM. Chromosome-specific nonrandom sister chromatid segregation during stem-cell division. Nature. 2013;498(7453):251-4.
