# Supplementary material for: RNA polymerase II-mediated rDNA transcription mediates rDNA copy number expansion in Drosophila
Source: PLoS Genet. 2024 May 17;20(5):e1011136. doi: 10.1371/journal.pgen.1011136 (PMC11139327; doi:10.1371/journal.pgen.1011136)
Supplement: Attachment — Submitted filename: Inrda-response 420.docx [file pgen.1011136.s007.docx]

Dear Scott,

Thank you so much for handling our manuscript, “RNA polymerase II-mediated rDNA transcription mediates rDNA copy number expansion in Drosophila”. We are pleased that the review comments are overall positive, and we thank you for the opportunity to revise the manuscript. In this revised version, we believe that we addressed almost all comments, and we hope that you will find the revised version is acceptable for publication in *PLoS Genetics*. In particular, we liked Reviewer #3’s comments about testing a-amanitin in indra-RNAi condition to see if IGS expression is mediated by pol II, which was indeed the case. We have added these new data into the revised version.

Best wishes,

Yukiko Yamashita

Point-by-point responses

Reviewer #1: The manuscript “RNA polymerase II-mediated rDNA transcription mediates rDNA copy number expansion in Drosophila” uses imaging of the germ stem cells of the testes to examine how repeat expansion of the rDNA is controlled. rDNA magnification is stimulated in genotypes with reduced numbers of rDNA copies. This group has previously identified a DNA-binding protein Indra that is required for magnification, and further characterize its role in magnification here. Overall this study shows clear data implicating repression of a promoter in the IGS of the rDNA unit by Indra in preventing repeat expansion. The authors link together expression of this IGS promoter, derepression of the R2 retroposon, and induced DNA damage as steps in repeat expansion specifically in the germ stem cells. This is an interesting study, but the significance of observations on nuclear distribution of RNAPII is unclear, and I have a few comments on exactly how these observations may fit together.

Lines 219-231 discuss the localization of RNAPII in the nucleus determined by immunofluorescence imaging. The authors argue that RNAPII is relocalized to rDNA genes at the periphery of the nucleolus, but this is a surprising result that is not clearly laid out in this section. It seems from Figure 5A vs 5B that there is a global relocalization of RNAPII to heterochromatin around the nucleolus, but does this really fit with the amount of repressed rDNA that is in these genotypes? This seems unlikely given how big these regions are and must encompass much more sequence than just rDNA. Could the authors give some sense of how much RNAPII they are detecting from image analysis, perhaps by analyzing the distribution of signal across the nucleus? Further, Figure 5E and 5J categorize nuclei based on interpretations of whether a nucleus has RNAPII at the nucleolus or not, but the authors should give some measure of how much this signal varies between cells.

(note that this reviewer is actually talking about Figure 6, not Figure 5). Please note that ‘perinuclear region’ is not entirely heterochromatin, although lots of heterochromatin are found near the nucleolus (and other fraction of heterochromatin goes to perinuclear/nuclear envelope compartment). We have added quantification on the image to show that indeed pol II is concentrated periphery of the nucleolus under low rDNA conditions and upon indra-RNAi, specifically in GSCs. Also this ‘nucleolar localization’ does not lead to depletion of pol II from the rest of the nucleus, as can be seen in images shown in Figure 6.

The authors use alpha-amanitin treatments to show that IGS transcription is RNAPII-dependent. Since they have observed that IGS transcription is correlated with R2 retroposon transcription, they go on to argue that both the IGS are transcribed by RNAPII (lines 233-242). This is surprising, because R2 is thought to be transcribed by RNAPI as part of a pre-rRNA. The model the authors lay out here and in the Discussion explicitly invoke a transcript originating in the IGS, but an alternative is that RNAPII-mediated transcription of the IGS promoter activates the adjacent rDNA RNAPI promoter. The authors might discuss these possibilities in the text or address the existence of long RNAPII transcript originating in the IGS. In many organisms including Drosophila and Arabidopsis short transcripts are produced from IGS regions, and the authors could discuss these results in a broader context.

Thank you for this suggestion. This reviewer raises a very important point, and indeed it is possible (none of our data exclude) that IGS-pol II simply invoke pol I-mediated transcription of R2-inserted rDNA copies. We have revised the text to include this point.

One caveat is the authors use a very high concentration of alpha-amanitin, and at these concentrations it may also inhibit RNAPIII.

Now we added 5S rRNA in situ hybridization (new Figure S3), which showed that 5S rRNA level did not change in our ex vivo culture condition, suggesting that RNAPIII is not affected in our condition.

Reviewer #2: In this manuscript the authors provide evidence that the indra protein is a negative regulator of rDNA magnification. Its an interesting study that advances the mechanistic insight into Indra, although some gaps in understanding still remain. I have provided some comments below for the authors’ consideration.

1. In the introduction the authors cite papers that say ERCs cause aging in yeast, but whether they are causal or just correlated has not been resolved-it would be good to acknowledge the controversy. The authors cite the original study but there have been several studies since and the situation may be more complicated, for example see Hotz et al., PNAS, 2022, Zylstra et al., PLoS Biology 2023

Thank you for the suggestion. Indeed, this issue has not been fully resolved in the field. We have revised the text to make the introduction about ERC more nuanced.

2. The asterisks in Figure 1 are tiny and difficult to see.

We have revised the Figure 1 to make the asterisk more visible.

3. How much is indra overexpressed in the overexpression experiments in Figure 2 and 4? Can the Indra antibody be used to estimate overexpression? If this was done previously, please provide the percent overexpression in the current manuscript with reference.

We have quantified Indra protein amount as a ratio between GSC and CySC (in new Fig S1). The most relevant comparison is between ‘animals with low rDNA CN’ and ‘animals with low rDNA CN with Indra overexpression’. In this comparison, we see that Indra is overexpressed ~1.7 times. We have added this figure as supplementary Figure (S1), and we modified the text to incorporate this information. (We used image-based analysis, because the expression level of Indra in GSCs is the most critical information here, and western blotting, which we have to use the cell extract that contains testicular somatic cells and non-GSC germ cells, will lose this most critical information).

4. Can the authors use their indra antibody to estimate the depletion in the RNAi condition? If this was done previously, please provide the percent depletion in the current manuscript with reference.

Indeed, we have previously reported indra RNAi, where we confirmed indra depletion by immunofluorescence staining (because somatic cells still maintain indra expression, and thus western blotting was not ideal) (Watase et al., 2022). We mentioned this in the revised manuscript.

5. Does indra overexpression over generations lead to the eventual loss of rDNA? Would the intermediate state eventually give way to the bobbed state?

Indeed, we have found Indra-OE leads to decline in fertility for about 5 generations, after which they recovered. We have consistently seen this trend (fertility decline and recovery) with various conditions that we know reduce rDNA CN, which we suspect to be similar to ‘ALT’ pathway of telomere lengthening. Because we do not know much about ‘alternative rDNA recovery mechanism’, we feel that this is a half-baked result. Although this ‘transgenerational decline in rDNA CN’ is an important aspect of future studies, we hope that the reviewer agrees that this is beyond the scope of the present study.

6. Can the authors estimate how many R2 containing versus non-containing repeats there are?

We have examined this with various wild type strains, and it varies a lot between strains as well as between X and Y chromosomes. It is somewhere between 30-60%. We do not understand yet whether some aspects of insertion (absolute insertion #, insertion frequency%, patterns of insertion (sparse vs. clustered), on which chromosome etc) might influence how R2 is expressed in response to rDNA CN changes. Thus, this number has to be taken as a grain of salt, but this number tells us at least it is not ‘mostly inserted’ or ‘mostly uninserted’.

7. Indra protein levels are examined. Although they are reduced in the low copy number flies, the amount of change doesn’t seem very substantial. How do the authors propose indra protein levels are regulated? Is the RNA regulated? This question plagued me throughout reading the manuscript. I don’t think the authors need to resolve it, but some speculation about how and why Indra levels are lower when copy number is lower would improve readability.

Indeed, this is a very important point, and we think that the regulation of indra protein amount must be at the heart of how cells transmit the information of rDNA CN to trigger magnification. Our data (in a separate study, which is being written up for publication now) show that indra RNA amount does not seem to change under the magnifying condition, thus we believe it must be post-transcriptional (e.g. translation, protein degradation). We briefly mentioned this in the revised Discussion (but we decided not to go into the details, because no existing evidence favors one way or the other, and speculation will not be able to go beyond ‘how protein amount may be regulated in general’.

8. Does Indra interact or colocalize with RNA Pol II?

Indra was originally identified as and IGS binding protein, and we have also shown that indra localizes to the nucleolus (Watase et al., 2022), which can also be shown throughout this manuscript. Thus, indra may localize at the periphery of the nucleolus when pol II is recruited there.

9. The authors should quantify the effects shown in Figure 7 not just for R2 but for IGS and ETS, to provide more evidence these are RNAP2 transcripts.

We have revised Figure 7 to include these quantifications.

10. Figure 6-it looks like RNAP2 is everywhere in the nucleus, and excluded from the nucleolus across all the conditions examined based on the images. How did the authors decide what qualified as signal at the nucleolar periphery.

We have revised Figure 6 according to this suggestion (which is detailed in the response to reviewer #1 comment above).

11. Number of observations should be provided for S2.
The S2 Figure (note that it is now S4 Fig) contained the number of observations within the panel.

Minor-
Probably better to use “containing” than “inserted”

We considered this, but the term ‘R2-inserted rDNA’ has been used by other researchers as well. Thus we decided to keep this as is.

Reviewer #3: In this study, Watase and Yamashita describe how rDNA magnification is regulated in male germline stem cells by zinc finger protein Indira to maintain germline immortality. The Yamashita lab has been steadily uncovering how metazoa regulate rDNA copy number using male germline stem cells from Drosophila as a model system. They previously discovered that rDNA copy number in under the control of Indira and a transposable element R2. R2 upregulation causes double strand breaks (DSBs), which then promotes homologous recombination to increase rDNA copy number. R2 does not have its own promoter but instead its transcription is under control of sequences in the intergenic spacers (IGS) in the rDNA repeats. However, the molecular mechanism of how R2 transcription is regulated was not fully clear.

Here they find that:
1. Low rDNA copy number promotes IGS transcription.
2. Indira represses IGS transcription under WT conditions.
3. Under low copy number levels of Indira decreases.
4. Downregulation of Indira promotes R2 transcription, which then promotes double strand breaks.
5. Indira promotes transcription of R2 via allowing access to pol II.

Conceptual advance: Here they find that the Zinc finger protein Indira that binds to IGS regulates R2 transcription that then promotes increase in copy number by promoting DSBs and recombination. In addition, their data suggests that the parts of the nucleolar compartment are privileged with sparing access to pol II that is under the control of Zinc finger protein Indira.

Overall, they describe a clear set of experiments in this well written manuscript. I have a few comments that should be addressed prior to publication:

1. Figure 1: It would be good to have a schematic of a testis with all the cell types they talk about in the supplementary data so this paper can be read by a broad audience that are the readers of PLOS Gen.

Thank you for the suggestion. We have added the cartoon of Drosophila spermatogenesis to Fig 1.

2. Figure 4B – they should mention the genotype. I understand this is an example, but it would be good to know if this what happens in Indira RNAi.

We have revised the figure according to this suggestion.

3. Figure 5 – again it would be good to know the genotype of 5A or see pictures of the other genotypes. Is there a pattern to this upregulation? In that do all GSCs in a single testis show an upregulation or is it stochastic under mutant conditions?

We have revised the figure according to this suggestion.

4. Figure 6— For me this is a weakest data they have in they have in the manuscript. The idea that Indira regulates RNA pol II levels to regulate IGS is gleaned from Figure 7. Figure 6 shows an almost a global increase in Pol II around the nucleolus. This can be due to many reasons including increased access to IGS sequence. I think this is supplementary data for Figure 7. If they want to include this data, it makes more sense to do a in situ of IGS and show there is an overlap.

Indra, Fibrillarin and IGS colocalizes in the nucleolus under normal and magnifying conditions. Therefore, Fibrillarin can be safely used as the proxy of IGS localization. We hope that the revision to Figure 6 (added quantification) made this figure stronger.

5. Figure 7—They should include an Indira RNAi + amanitin condition to bolster their model.

This is a great comment. Thank you for this great suggestion. We have added this experiment, which is now included in Figure 7.

Reviewer #4: This is a review of Manuscript PGENETICS-D-24-00048, “RNA polymerase II-mediated rDNA transcription mediates rDNA copy number expansion in Drosophila,” by Watase and Yamashita.

The manuscript purports to show that the indra gene product binds to rDNA and mediates rDNA magnification in Drosophila. Specifically, the authors make 7 claims:

1 - Indra is a negative regulator of the expression of the Intergenic Spacer in the rDNA
2 - Indra protein level is tightly regulated in Germ Cells
3 - Indra protein regulation is regulated by rDNA multiplicity
4 - IGS expression is thereby controlled by rDNA copy number
5 - RNA Polymerase II is responsible for IGS expression
6 - IGS expression reads through to R2-laden rDNA cistrons
7 - RNA polymerase is responsible for R2 expression

Overall, I find the work described here to be superficial, poorly controlled, and many of the data simply not believable. The central claims are generally unsupported by the data.

We appreciate that the editor recognized the negative tone of this reviewer. While this reviewer’s comments are indeed mostly not constructive, we feel that it is important that we provide the response to some of the issues this reviewer raises because some criticisms are unfounded, and we want to leave a record (although PLOS Genetics does not publish the reviews) to state that our methods and approaches are not compromised in rigor as implied by this reviewer. In some instances, it appears that this reviewer provides partial information to construct their criticisms on our manuscript, which we felt misleading. Thus, we wanted to clarify those issues.

Major Concerns

1 - The assays seem to rely on quantitative in situ hybridization, which are not known to be quantitative. The nucleolus and rDNA within it are dynamic in terms of condensation and probe accessibility, as well as copy number. The data in Figure 1E, 2D, etc, even backed up with “best pictures,” are not believable without some form of independent quantification, and some evidence that the FISH signal is sensitive (i.e., zero signal is zero rDNA), proportional (i.e., N% increase in signal is N% increase in rDNA), controlled for bleaching, etc. I am made more skeptical by the images in Figure 2 since according to the quantification, the images shown are selected to show a more extreme effect size than the data generally.

More classical methods of in situ hybridization that involves signal amplification are indeed not quantitative, but thanks to recent development of single molecule FISH, fluorescent oligo probes etc makes it highly quantitative. It may not be as quantitative as biochemistry, but it is a widely accepted method that are recognized to be quantitative.

2 - Much relies on the statistical difference between conditions, but no attention is paid to effect size. In most cases (e.g., Figure 3F), the difference is detectable by Mann Whitney U-test, but most of the data overlap. There is no explanation why identical intensities (in the two conditions) would yield different outcomes. Simply detecting a difference in average is not enough to explain the phenomenology. The error bars appear to be standard error of the mean (although the authors never mention what they represent), which for population data is the incorrect statistic; standard deviation should be used.

We find the comment ‘the difference is detectable by Mann Whitney U-test, but most of the data overlap’ is as if this reviewer denies the use of statistics as a whole. The data that do not overlap do not require statistics (‘human is larger than ants’), but most of meaningful differences come with overlapping data (‘human males are taller than human females’). We used Mann-Whitney test for the dot graphs, because the data set in each data group does not necessary follow Gaussian distribution: student-t test is only applicable when dot distribution in two groups (control vs. experiment) follows Gaussian distribution. We believe Mann-Whitney test is most suitable here. We have added the info on error bars throughout the figure legends.

3 - The experiments showing effects on rDNA expression (or R2 expression) as a consequence of altering indra are without necessary controls. In no experiment do the authors show how much indra overexpression there is, or how much indra is reduced in RNAi conditions. They do not show any changes in overall or per-IGS binding by indra. For that matter, the amount (variation) of indra is not measured, compromising one of their central claims.

We have responded regarding the amount of indra overexpression, which is in response to other reviewer comments.

4 - The data in Figure 3 do not appear normalized to rDNA copy number. This is an oversight, since the authors claims that indra binds to IGS, so is expected to be reduced in those cases where IGS copy number is lower. It is not possible without quantification of both protein and rDNA copy number to confirm that binding per IGS is altered. Hence, these data cannot be used (in this form) to support their hypothesis of lower indra binding. Further, the repeat number of the IGS units is not consistent between rDNA IGS copies. Quantifying rDNA by a bobbed phenotype (as the authors do) ignores the R1- and R2-inserted components, and further ignores the variable repeat structure of the IGS. To make the claims they make, the authors must quantify the IGS repeats that act as the polII promoter element.

This is a good point that we have wondered for a very long time. If we can ‘perfectly normalize’ this, we are likely able to figure out the actual underlying mechanism of how indra amount is connected to rDNA CN counting mechanism. For now, we don’t have that level of understanding, but we believe we have enough to claim that indra amount goes down when rDNA CN goes down, beyond ‘just proportional to CN’. In ‘normal rDNA CN’ condition, GSC and GB/SG are expected to have equal rDNA CN (because USCE is not triggered), yet GSC has more indra. Then under ‘low rDNA CN’ condition, where GSC likely has more rDNA CN than GBs (this was demonstrated in our previous paper (Watase et al., 2022)), indra amount is the same between GSC and GB, which should indicate that GSC has less indra per rDNA copy. By combining data from other figures, we believe that our claims are justified.

5 - The image in S1 do not demonstrate an X-Y exchange. Certainly a better image can be produced, especially once such an X-Y exchange is isolated genetically into a strain.

Mitotic XY recombination likely leads to cell death (indra-RNAi leads to rapid cell death). We also know that such rapid cell death can be rescued by removing rDNA from either X or Y chromosome. (that is, indra^RNAi^; bb^158^/Y or indra^RNAi^ ; X/Ybb, are considerably better, which suggests indeed XY recombination in indra^RNAi^ leads germ cell death). However, we do not think that this much of explanation is necessary in the context of our claim surrounding Figure S1 (now Fig S2).

6 - The FISH in Figure S2 are also not believable. FISH within the rDNA often show bends, flops, areas of less condensation, etc. These images, in my experience, show the regular variety in rDNA. Notably, here, the “wild-type” images are of highly condensed rDNA arrays, and the images from “indra” show decondensed chromosomes, judging even by the autosomes visible in the spreads. The image in C’ dos not eve appear associated with a chromosome.

We find this comment to be one of those that are unnecessarily critical. And this is not just FISH, but co-FISH that visualizes two sister chromatids that are next to each other, and we never found any situation where ‘less condensed DNA’ results in cross-checker patterns of ‘red’ vs. ‘blue’ strands.

7 - The involvement of RNA polII by IF is simply not believable. The perinucleolar enrichment is not convincing without a careful analysis of polII localization throughout the cell cycle, in different strains, etc. The criteria for the data in 6E are never described: how can one do statistical analysis on subjective decisions whether polII is enriched? As far as I know, and as far as the authors cite, polII has not been shown to transcribe the IGS in Drosophila. Basic characterization of the hypothesized polII promoter is a necessary part of the author’s model.

We have addressed this issue in response to other reviewers to the extent that is reasonable.

8 - The treatment with amanitin (for two hours) is subject to secondary effects. The authors cannot claim that this is a direct inhibition of PolII at the rDNA instead of secondary effects based on blocking all mRNA production. Such treatment will alter the cell cycle, place the cells into stress, reduce concentrations of proteins with relatively high turnover, etc. None of those viable alternatives are tested.

This goes to any kind of inhibitor treatments, but at least 2 hours are not the time window cells start dying due to the lack of essential mRNA not producing essential proteins.

9 - Much could be resolved by more careful analysis. Single-cell chIP (with indra, polII, etc) would directly test some of the claims that the authors make. Frankly, the idea that Drosophila evolved indra to allow polII to read through the IGS and into the rDNA in order to use a retrotransposon to amplify the rDNA is an extraordinary claim. Much of that is directly testable – for example a cloned cDNA from the IGS through to R2 – and the authors should be compelled to do so before making such a claim.

IGS is a known promoter for Pol II. It has been already known R2-inserted vs. -uninserted rDNA copies are differentially regulated (inserted copies are silenced under normal conditions). It has been already known that R2 is transcribed as ‘read through’ of rDNA without its own promoter. Combining all these, our claim is only integrating all known knowledge, and simply placing indra in such a context. Our claim is nothing like ‘Drosophila evolved Indra to transcribe R2’ as this reviewer described.

10 - The authors should justify using the Mann-Whitney U-test, instead of ANOVA or t-tests. As far as I know, the MWU is primarily used for categorical data, which these are not.

See our comments to this reviwer above.

11 - It seems that the entire genome is damaged in Figure 4B. This seems to be well outside of the rDNA foci (judging by Figures 1-3), calling this analysis into question.

This is well known and reproduced by many researchers (outside our labs too). For unknown reason, gH2Av signal becomes ‘almost entirely nuclear’ in male germ cells (with the same antibody, the same conditions and the same sets of hands, we see punctate gH2Av foci in other cell types, including.

Some statements are purely speculative and do not belong in the Results section (e.g., lines 164-166).

Others – such as how indra is regulated (protein stability? translation? transcription?), and how rDNA copy number is ascertained by that regulatory mechanism – that seem fundamental to the claims in the paper are not mentioned.

The linkage between aging and rDNA is limited to yeast, so the references to aging seem misplaced here.

These comments are not constructive in any ways. Where to insert speculation is up to authors, as far as it is clear what is speculation and what is conclusions. Some speculation is not worth the whole section in the Discussion, and we do not see any problem to insert a short speculation in result section, when we clearly state that it is ‘implication’ but not ‘conclusion’..

The regulation of indra by rDNA CN is indeed our interest, but clearly outside the scope of this current study.

Additionally, there are some problems with how the authors treat the literature. Sharyn Endow showed USCE as the mechanism of rDNA magnification, and her results did not reveal a strand bias since both products were recovered. Regardless of the latter, it is imperative that proper credit be given to Endow and her laboratory. Futher, Komma and Endow demonstrated magnification in females, so the author’s statement that rDNA magnification is limited to the male germ line is incorrect. This is reviewed in the Hawley and Marcus paper that the authors cite.

We are fully aware of their literature (Endow, Marcus/Hawley), so is this reviewer. That said, this reviewer likely knows more than they mention in this comment that Endow and Hawley/Marcus papers are NOT inconsistent at all with the work described in this paper. Regarding the strand bias, our recent paper (Nelson et al., 2023) fully explains how USCE and strand bias leads to ‘many magnified’ males and ‘fewer worsened’ males. If this reviewer is mentioning the lack of strand bias during meiotic magnification by Endow, it is already shown that meiotic magnification is distinct from mitotic magnification, and this reviewer is likely aware of such distinction demonstrated by Hawley and Tartof (Hawley and Tartof, 1985), and we wonder why this reviewer leaves out this critical information (likely intentionally) in constructing criticisms on our work. Another example of ‘left out information’ is this reviewer’s mentioning of magnification in females, which is limited to an unusual situation of females containing Y chromosome (XXY).

**References**

Hawley, R.S., Tartof, K.D., 1985. A two-stage model for the control of rDNA magnification. Genetics 109, 691-700.

Nelson, J.O., Kumon, T., Yamashita, Y.M., 2023. rDNA magnification is a unique feature of germline stem cells. Proc Natl Acad Sci U S A 120, e2314440120.

Watase, G.J., Nelson, J.O., Yamashita, Y.M., 2022. Nonrandom sister chromatid segregation mediates rDNA copy number maintenance in Drosophila. Sci Adv 8, eabo4443.
